# The transformative impact of artificial intelligence on periodontal care: A review

**DOI:** 10.6026/973206300220369

**Published:** 2026-01-31

**Authors:** Jaideep Mahendra, Devadharshini Chandrasekar, Roshini Govindarajan, Pavithra H Dave, Sajid T Hussain, Muskan Bedi

**Affiliations:** 1 Department of Periodontics, Meenakshi Ammal Dental College and Hospital, Meenakshi Academy of Higher Education and Research, Chennai, Tamil Nadu, India; 2 Department of Periodontology and Implantology, Bharath University, Sree Balaji Dental College and Hospital, Chennai, Tamil Nadu, India; 3 Department of Medicine, Sri Ramachandra Medical College and Research Institute, Chennai, Tamil Nadu, India

**Keywords:** Artificial intelligence (AI), electronic brain, machine learning, neural network, dental care

## Abstract

Artificial intelligence (AI) is emerging as a transformative force in periodontal care, reshaping traditional diagnostic, preventive and therapeutic paradigms. Recent advances in algorithms, computational power and access to large-scale digital datasets have enabled the development of sophisticated AI and machine learning (ML) models capable of analyzing complex clinical, radiographic and biological data. These technologies support clinicians in early diagnosis, risk assessment, personalized treatment planning and prediction of disease progression. By integrating AI-driven analytics with the principles of evidence-based dentistry (EBD), periodontal care is transitioning toward more precise, efficient and patient-centered approaches. Thus, we show the evolution of AI in periodontology, its current and emerging clinical applications and its potential to enhance decision-making, optimize outcomes and redefine the future landscape of periodontal practice.

## Background:

Artificial intelligence (AI) is revolutionizing healthcare by enabling machines to perform tasks that were previously limited to human intelligence. This technology holds significant promise by facilitating fewer unnecessary interventions, enhancing clinical decision- making, reducing postoperative complications and ultimately improving patient quality of life. In dentistry, AI is increasingly being utilized for diagnostic accuracy, treatment prediction and differentiation between normal and pathological structures [1]. Artificial intelligence, as a discipline of computer science, seeks to design and develop intelligent systems, most commonly in the form of software programs. It may be described as a sequence of actions directed toward accomplishing a specific task. Historically, AI systems relied on hand-crafted rules, requiring extensive engineering, subject-matter expertise and domain-specific knowledge. For example, in medical imaging, an algorithm could be designed to detect abnormal growths or variations in tissue appearance [2]. In the field of periodontology, AI has shown remarkable potential in enhancing diagnostic precision, assessing disease severity and predicting treatment outcomes. Machine learning algorithms can analyze clinical and radiographic data to identify subtle patterns of periodontal destruction that may not be easily detectable by the human eye. Moreover, AI-driven tools support clinicians in risk assessment, treatment planning and long-term disease monitoring, thereby promoting a more personalized and preventive approach to periodontal care. Therefore, it is of interest to emphasize the emerging applications of AI in dentistry, with particular focus on periodontology, highlighting its role in diagnosis, evidence-based decision-making and therapeutic prediction, while also addressing the current challenges and barriers to clinical integration.

## Fundamentals of artificial intelligence and machine learning:

Modern artificial intelligence is generally divided into two types: narrow AI and general AI. Narrow AI includes a range of specialized approaches, such as machine learning models and expert-based systems [3]. Within the field of artificial intelligence (AI), machine learning [ML] enables computers to identify patterns and make decisions without relying on explicit programming or prior knowledge. Through training, algorithms are exposed to large datasets, progressively adjusting internal parameters until accurate outputs are achieved, thereby allowing them to generalize and analyze new data effectively [4]. Deep learning (DL), an advanced subset of ML, extends this process by creating hierarchical representations of data through layered networks, enabling recognition of increasingly complex patterns that simulate human cognitive processes [5]. Artificial neural networks (ANNs), composed of interconnected computational units or "neurons," form the basis of these systems, with input, hidden and output layers working collectively to generate predictions. Depending on their depth, these networks may be classified as shallow neural networks (SNNs) or deep neural networks (DNNs), where hidden layers and calibrated weights determine the accuracy and adaptability of outputs [6]. Among ANN architectures, convolutional neural networks (CNNs) are widely applied in medicine and dentistry due to their superior performance in image recognition. By applying convolutional filters in sliding windows across digital inputs, CNNs can efficiently detect and classify anatomical structures. In periodontology, CNNs have demonstrated promising applications in the detection of alveolar bone loss on radiographs, identification of periodontal defects and differentiation of disease severity stages and prediction of treatment outcomes. Furthermore, AI-driven models have been employed to assist in risk assessment for periodontitis progression, automate periodontal charting and evaluate regenerative therapy outcomes through image-based analysis. These advancements highlight the transformative role of AI in enhancing diagnostic precision, treatment planning and prognostic accuracy in periodontal care.

## Applications of artificial intelligence in dentistry:

## AI in dental imaging and diagnosis:

Convolutional neural networks [CNNs] have demonstrated promise in their capacity to identify and detect anatomical features. For example, some have been trained to identify and label teeth from periapical radiographs. CNNs have demonstrated a precision rate of 95.8-99.45% in tooth recognition and identification, almost matching the 99.98% precision rate of clinical specialists [7]. AI as a tool in dentistry: Artificial intelligence (AI) has shown remarkable advancement in enhancing practicability and precision in the field of dentistry. Among the various AI approaches, convolutional neural networks (CNNs) have been particularly effective in the diagnosis and detection of dental caries. Deep CNN models trained on large radiographic datasets, including periapical, bitewing and panoramic images, have demonstrated strong diagnostic performance in identifying carious lesions. In a comprehensive review of AI-based radiographic caries detection, reported that CNNs achieved high sensitivity (72-98%) and specificity (up to 98%), with overall diagnostic accuracy frequently exceeding that of human examiners. These systems not only improve the speed and consistency of caries detection but also significantly reduce interobserver variability and diagnostic subjectivity. Due to their high efficiency and potential to enhance diagnostic reliability, deep CNNs are considered among the most successful AI methods currently applied in dental imaging and diagnostics [8].

## AI in orthodontic treatment planning:

Carefully planning orthodontic treatments is necessary to provide patients with predictable results. On the other hand, it is not unusual for the orthodontic treatment plan to include tooth extractions. Because extractions are irreversible, it is critical to ensure that the most appropriate clinical decision is made before initiating treatment. Artificial intelligence (AI), particularly through artificial neural networks (ANNs), has emerged as a valuable decision-support tool in this context. ANNs have been developed to assist clinicians in determining whether tooth extractions are required in cases of malocclusion by analyzing multiple clinical and cephalometric indicators. Recent studies have evaluated the performance of various AI models in predicting extraction decisions in orthodontics. The pooled results indicated that AI algorithms achieved a mean diagnostic accuracy of approximately 87%, with sensitivity around 84% and specificity near 89%, highlighting their strong predictive capability. These findings indicate that ANN-based models can replicate expert clinical reasoning, providing objective and data-driven support that enhances diagnostic consistency and efficiency in orthodontic treatment planning [9].

## Artificial Intelligence in Periodontology:

## Diagnosis and risk prediction:

Artificial intelligence (AI) has emerged as a powerful tool in periodontology, offering advanced methods for the diagnosis and prediction of periodontally compromised teeth (PCTs) as well as differentiation between forms of periodontal disease. In addition to diagnostic applications, AI enables the development of personalized risk profiles by integrating patient-specific demographic, genetic and clinical data. Machine learning models can predict the periodontal disease progression, enabling clinicians to adopt preventive interventions to reduce further tissue damage [10]. Recent studies have demonstrated that deep learning algorithms, particularly convolutional neural networks (CNNs), can analyze large sets of radiographic data to identify periodontal bone loss and predict tooth prognosis with high accuracy. For instance, a study employing a deep-learning ensemble model on approximately 8,000 periapical radiographs reported overall diagnostic accuracy of around 90%, with periodontal bone loss detection reaching 97% [11].

## Differentiation of periodontal diseases:

Beyond radiographic analysis, AI models, including artificial neural networks (ANNs), have also been applied to integrate clinical, immunologic and hematologic parameters such as interleukin levels, leukocyte counts and other blood biomarkers-to distinguish between aggressive periodontitis (AgP) and chronic periodontitis (CP) with high precision. These findings highlight AI's potential to enhance diagnostic precision, improve treatment planning, support early intervention and facilitate personalized and predictive approaches in periodontology [12].

## AI in tooth salvage and endodontics:

Salvaging teeth using AI: While mandibular molar root canal designs are generally similar, there are a few uncommon variances that can happen. Cone-beam computed tomography [CBCT], which minimises treatment failures attributable to morphological differences, has become the gold standard for optimising the clinical outcomes of endodontic therapy. But because CBCT exposes patients to more radiation than traditional radiography, it is not used frequently. A recent study evaluated root canal configurations of maxillary and mandibular first molars using CBCT and highlighted the prevalence of complex canal anatomy, supporting the need for AI-assisted diagnostic tools to reduce missed canals and improve treatment outcomes. Despite achieving accuracy rates up to 86.9%, CNN-based systems still face challenges for clinical integration, including time-consuming manual image segmentation and the need for appropriately sized, high- quality images to ensure reliable classification [13].

## AI in oral lesion detection and diagnostic sciences:

Application of AI in understanding lesion sciences: Recognizing and diagnosing oral health issues is essential in dental practices since early intervention frequently results in a better prognosis. Accurate diagnosis and appropriate treatment are crucial because certain oral lesions may have malignant or precancerous origins. CNN has proven to be a useful technique for diagnosing head and neck cancer lesions. A study using hyperspectral imaging combined with deep CNNs demonstrated the ability to distinguish squamous cell carcinoma from normal tissues with approximately 81% accuracy, sensitivity and specificity, highlighting the potential of AI for non- invasive, real-time lesion identification and surgical guidance [14]. Additionally, a study employing CNNs to differentiate between ameloblastomas and odontogenic keratocysts using cone-beam computed tomography (CBCT) images achieved sensitivity of 87.2%, specificity of 82.1% and accuracy of 84.6%, outperforming both senior and junior oral and maxillofacial surgeons. These findings underscore the growing role of AI in enhancing diagnostic precision and supporting early intervention in oral health care [15].

## Artificial intelligence-challenges and strength:

AI in healthcare faces challenges in managing and exchanging clinical data, as it requires patient data for training and validation. Privacy and confidentiality must be protected, as the healthcare community is skeptical about safe data exchange. AI systems pose safety concerns and require regulation mechanisms. The US Food and Drug Administration established the "Software as Medical Device" drug category to control patient safety. AI systems also raise ambiguous accountability, as the developer or expert may be at fault. This poses ethical and legal order issues, potentially threatening the legal system. Transparency in AI data and algorithms is crucial for accurate predictions. Inadequate data labelling can lead to subpar outcomes, limiting the effectiveness of AI systems. Medical practitioners must understand and defend AI predictions and significant advancements are needed for neural networks to provide transparent clinical diagnosis or treatment suggestions [16, 17].

## Conclusion and future prospects:

Artificial intelligence in dentistry is not a future replacement, but rather a helpful adjunct for specialists and dentists. To maintain human autonomy, AI should be safely integrated and dental schools need to provide continuous education and dental training for successful integration. Artificial intelligence [AI] is revolutionizing augmented and virtual reality, transforming surgical planning and learning. Mixed reality, combining virtual and generative AI, offers promising solutions for dental specialties, presenting significant potential for the growth of dentistry.
